# Correction to “Curcumin induces ferroptosis in non‐small‐cell lung cancer via activating autophagy”

**DOI:** 10.1111/1759-7714.15298

**Published:** 2024-04-05

Tang X , Ding H , Liang M , Chen X. , Yan Y. , Wan N. , Chen Q. , Zhang J. , Cao J. Curcumin induces ferroptosis in non‐small‐cell lung cancer via activating autophagy. Thorac Cancer 2021;12:1219–1230. 10.1111/1759-7714.13904 33656766 PMC8046146

In the original version of this paper, errors were identified in Figures 5c and 6c. The rest of the content in the images remains unchanged, as well as the interpretation of the results and conclusions. The corrected graphical panels are included below.

**FIGURE 5:**
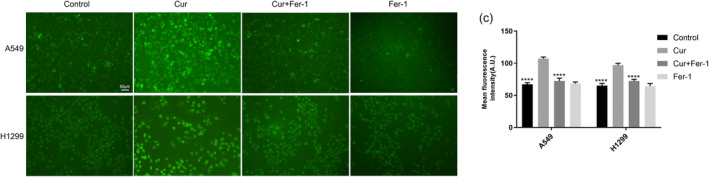
(c) and corresponding statistical charts.

**FIGURE 6:**
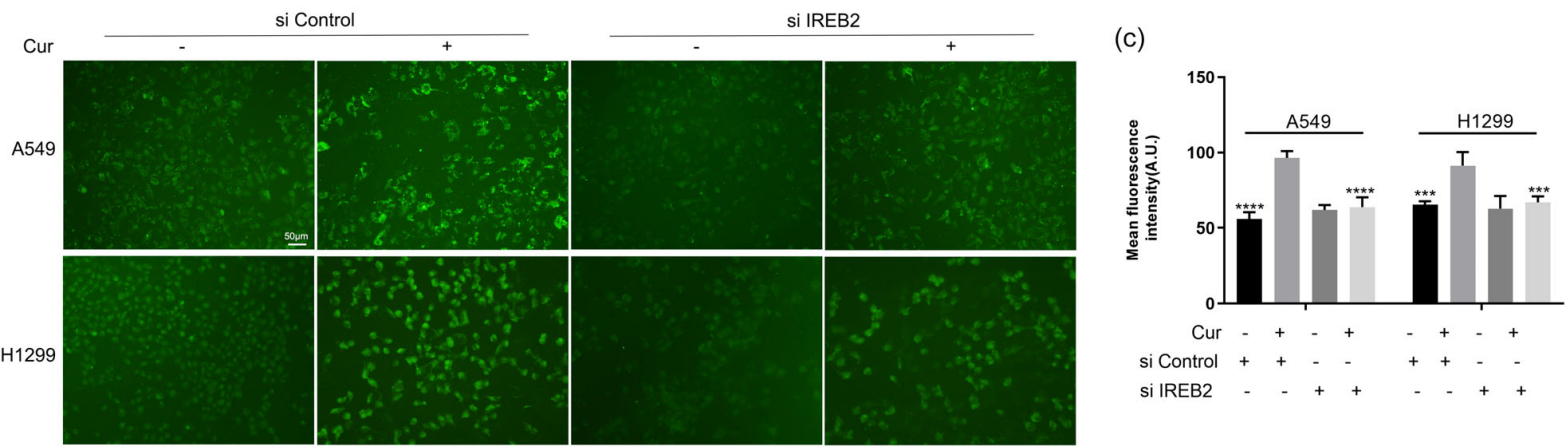
(c) and corresponding statistical charts.

We apologize for these errors.

